# Detection of Cerebral Hemorrhage in Rabbits by Time-Difference Magnetic Inductive Phase Shift Spectroscopy

**DOI:** 10.1371/journal.pone.0128127

**Published:** 2015-05-22

**Authors:** Wencai Pan, Qingguang Yan, Mingxin Qin, Gui Jin, Jian Sun, Xu Ning, Wei Zhuang, Bin Peng, Gen Li

**Affiliations:** 1 College of Biomedical Engineering, Third Military Medical University, Chongqing, China; 2 State Key Laboratory of Trauma, Burns and Combined Injury, Daping Hospital, Surgery Institute of the Third Military Medical University, Chongqing, China; 3 Department of Biomedical Engineering, Chongqing University, Chongqing, China; CNRS, FRANCE

## Abstract

Cerebral hemorrhage, a difficult issue in clinical practice, is often detected and studied with computed tomography (CT), magnetic resonance imaging (MRI), and positron emission tomography (PET). However, these expensive devices are not readily available in economically underdeveloped regions, and hence are unable to provide bedside and emergency on-site monitoring. The magnetic inductive phase shift (MIPS) is an emerging technology that may become a new tool to detect cerebral hemorrhage and to serve as an inexpensive partial substitute to medical imaging. In order to study a wider band of cerebral hemorrhage MIPS and to provide more useful information for measuring cerebral hemorrhage, we established a cerebral hemorrhage magnetic induction phase shift spectroscopy (MIPSS) detection system. Thirteen rabbits with five cerebral hemorrhage states were studied using a single coil-coil within a 1 MHz-200 MHz frequency range in linear sweep. A feature band (FB) with the highest detection sensitivity and the greatest stability was selected for further analysis and processing. In addition, a maximum conductivity cerebrospinal fluid (CSF) MRI was performed to verify and interpret the MIPSS result. The average phase shift change induced by a 3 ml injection of autologous blood under FB was -7.7503° ± 1.4204°, which was considerably larger than our previous work. Data analysis with a non-parametric statistical Friedman M test showed that in the FB, MIPSS could distinguish the five states of cerebral hemorrhage in rabbits, with a statistical significance of p<0.05. A B-F distribution profile was designed according to the MIPSS under FB that can provide instantaneous diagnostic information about the cerebral hemorrhage severity from a single set of measurements. The results illustrate that the MIPSS detection method is able to provide a new possibility for real-time monitoring and diagnosis of the severity of cerebral hemorrhage.

## Introduction

Cerebral hemorrhage is the second-largest cause of strokes, accounting for 10 to 15 percent of all stroke patients [1]. It is accompanied by high incidence, high morbidity, high mortality and heavy economic burden. Van et al. reported that the overall incidence of cerebral hemorrhage was 24.6 per 100,000 from 1980 to 2008, and that the incidence increases with age [2]. Cerebral hemorrhage poses a serious threat to human health and life, including primary and secondary brain damage [1]. Nerve injury, caused by hematoma proliferation, is mainly associated with hematoma-induced physical injury at 0–4 h after cerebral hemorrhage [3]. Therefore, real-time monitoring and assessment of the severity and developmental course of cerebral hemorrhage is a key to its treatment.

Currently, many detection methods are used to measure intracranial hemorrhage, including direct measurement of intracranial pressure (ICP) [4], computer tomography (CT), positron emission tomography (PET) and magnetic resonance imaging (MRI) [5]. In the ICP monitoring method, the sensor is inserted into the brain, which may cause injury and infection. The CT, PET and MRI and other imaging methods have the shortcomings of large size and the inability to provide bedside and emergency on-site monitoring. Moreover, these devices are very expensive, thus limiting their use in economically underdeveloped regions.

The Magnetic Induction Phase Shift (MIPS), which is based upon detecting characteristic parameters such as the conductivity of diseased tissue, is non-contact, non-invasive, inexpensive, small and able to maintain continuous bedside monitoring, and is a new method for detecting cerebral hemorrhage [6–10]. There are two kinds of difference techniques in MIPS research—the time-difference method and the frequency-difference method. The time-difference method is used to detect the phase shift differences between before and after a simulated lesion occurs, and can be used for monitoring purposes [9, 11, 12]. The frequency-difference method is self-referencing and can be performed in a short time, providing instantaneous information of cerebral hemorrhage [13].

When a traditional single excitation coil and a single receiving coil (single coil—coil) is used to detect cerebral hemorrhage, the magnetic field sensed by the receiving coil can be divided into three parts: the primary field generated by excitation source, the perturbation field generated by other brain tissues, and the perturbation field of interest generated by the bleeding or ischemic infarction sites. Since the conductivity of the biological tissue is very small (σ < 3 s/m), the magnetic field disturbance generated by the brain tissues is very weak (for example, the perturbation field accounts for only 1% of the main magnetic field at the frequency of 10 MHz [6, 14]). Moreover, the target field accounts for a small proportion of the entire perturbation magnetic field. Therefore, the signal of interest is extremely weak. In order to improve the measurement sensitivity, both the primary field and the perturbation field generated by other brain tissues must be canceled.

In our previous work, we conducted simulation studies on magnetic induction tomography (MIT) measurement system [15], designed a new type excitation source, implemented the optimal excitation coil for MIT [16], adopted the MIPS method for detecting normal and edema nerve cells [17], carried out experimental study on simulated cerebral edema and cerebral hemorrhage detection with MIPS [18–19], and detected the MIPS of cerebral hemorrhage in rabbits [20]. These works demonstrated the feasibility of detecting cerebral hemorrhage with the MIPS method. However, the detection sensitivity of cerebral hemorrhage was very limited. For better detection results, we re-established a new magnetic induction phase detection system, which could achieve phase noise as low as 6 m° and a 4-hour phase drift as low as 30 m° at 21.4 MHz [21], and studied the MIPS of cerebral hemorrhage in rabbits with a single coil-coil [22]. Furthermore, we designed a symmetric cancellation-type sensor detection system based on the symmetry between the two brain hemispheres so as to offset the interference of the primary field and the perturbation field generated by other brain tissues [23]. Notwithstanding some improvement in detection sensitivity, we were unable to achieve a desired result.

In the previous work (our team and other teams), we found that the excitation signals used were a single or a limited number of frequencies. Thus, we were unable to learn the MIPS information of other frequencies and could not select the frequency band with an optimal sensitivity for the experimental analysis. In order to study a wider band of cerebral hemorrhage MIPS, we established a cerebral hemorrhage magnetic induction phase shift spectroscopy (MIPSS) detection system. Thirteen rabbits with five cerebral hemorrhage states were studied by a single coil-coil within a 1 MHz-200 MHz frequency range in linear sweep. A feature band (FB) with an optimal detection sensitivity and stability within the entire band was determined. Then the data of MIPSS of five cerebral hemorrhage states under FB were analyzed. The results showed that the MIPSS of cerebral hemorrhage under FB has high detection sensitivity, thus providing the condition for subsequent data processing.

## Materials and Methods

### 1. Detection Principle

Griffiths et al. reported that the sinusoidal excitation signal (*Ie* ^*jωt*^) emits the sinusoidal alternating main magnetic field by the excitation coil (**B**). The main magnetic field introduced an induction current into the brain tissue, and the current caused a perturbation in the magnetic field (Δ**B**). The induction coil then received the magnetic field (**B** + Δ**B**), which was obtained by adding the main magnetic field to the perturbation magnetic field [6–7]. The phase shift difference (Δθ) was produced between the received signal and the excitation signal. The size of the phase shift difference was associated with the brain tissue conductivity and the frequency of the excitation signal.

**Fig 1:**
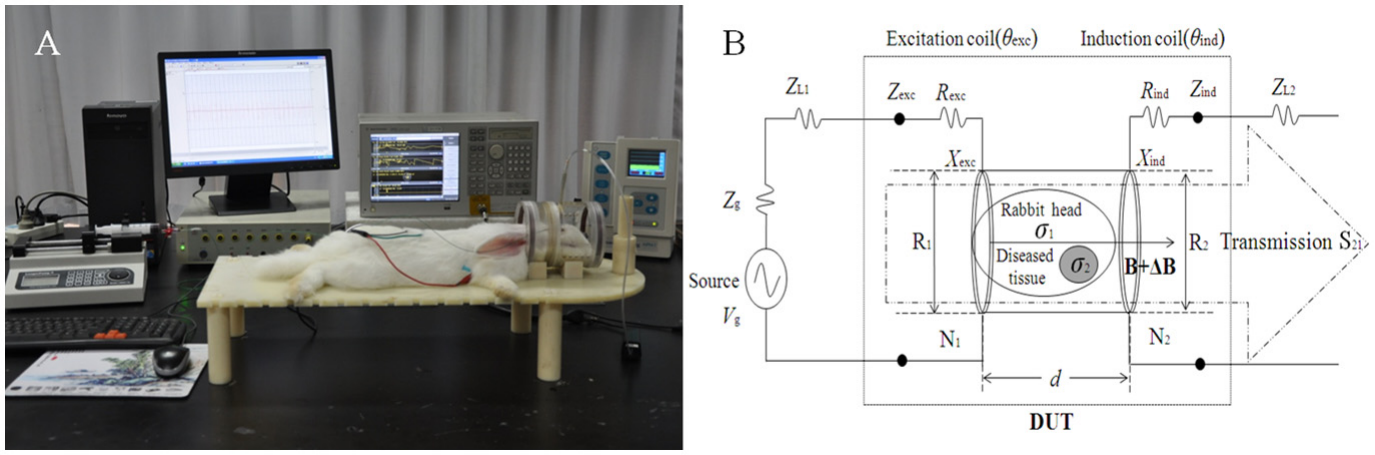
Photograph of experimental setup (A) and equivalent circuit (B) of cerebral hemorrhage magnetic induction detection system. The equivalent circuit is an inductive coupling of two RL series elements by a conductive ellipsoid media. Zg represents source impedance. ZL represents transmission line impedance. Zexc (Zind) represents coil impedance which is composited by the resistance Rexc (Rind) and the inductance Xexc (Xind).

An equivalent circuit diagram of the magnetic induction detection for cerebral hemorrhage in rabbits is shown in Fig 1B. The phase shift difference Δθ caused by brain lesions was obtained by subtracting the phase shift of the induction coil signal from the phase shift of the excitation coil signal. Its formula was as follows:

$$\Delta \theta =\theta_{ind}-\theta_{exc}$$ (1)

This study represented a relative phase shift difference which was obtained by subtracting the phase shift induced by injecting different volumes of autoblood into the rabbits’ head from the baseline phase shift.

### 2. Experimental System

The cerebral hemorrhage magnetic induction detection system, shown in Fig 1A, was composed of two modules: a RF vector network analyzer (Agilent E5061A), and a two-coil structure containing a rabbit model of cerebral hemorrhage.

In this study, the RF vector network analyzer, a two-port network, was used to measure the amplitude and phase between the excitation coil signal and the induction coil signal. The signals generated by the excitation coil (port 1) were sent to the induction coil (port 2). During the transmission process, they were expressed as transmission parameters S_21_. Hence the transmission parameters S_21_were the channel required for measurement. The vector network analyzer was set to simultaneously measure the amplitude and phase information of the transmission parameters S_21_. The output power of the signal source was 10 dBm. The excitation signal frequency ranged from 1 MHz to 200 MHz, and its work mode was set as a linear scanning run mode.

The coils were composed of the excitation coil and the induction coil, with radii R_1_ = R_2_ = 5.2 cm. The two coils were twisted with a 1 mm-diameter copper wire in a special plexiglass mold, with N_1_ = N_2_ = 10. The two coils were symmetrical, and were placed in parallel, with distance of d = 10.5 cm. A rabbit’s head was placed in the lower middle of the two coils. The two coils were connected to a vector network analyzer by a high-frequency coaxial line.

The anesthetized rabbit was fixed on a non-magnetic material table. A 20 ml plastic syringe containing 4 ml of fresh autologous blood was connected to the rabbit brain with a catheter. The injection rate was controlled by using an electronic syringe pump. A thermometer and hygrometer were used for real time display of the temperature and humidity. An intracranial pressure monitor (CAMINO MPM-1, INTERGRA, USA) was used to measure the ICP of the rabbits. A multi-channel physiological signal acquisition instrument (RM6280C, Chengdu Instrument Factory, China) was used to continuously monitor the rabbit’s ECG and heart rate variability (HRV).

### 3. Experimental Design

#### 3.1. Cerebral hemorrhage model in rabbits

Thirteen rabbits (2.3 ± 0.2 kg) in the MIPSS measurement group and five rabbits (2.2 ± 0.2 kg) in the MRI measurement group were obtained from DaPing Hospital, ChongQing, China. They received humane care from a properly trained professional in compliance with the Management Regulations for Laboratory Animals, formulated and published by the State Scientific and Technological Commission of China. The Animal Experiments and Ethics Committee of Third Military Medical University approved all experimental protocols, and care of the animals was carried out in accordance with the Declaration of Helsinki and IASP guidelines [24, 25].

**Fig 2:**
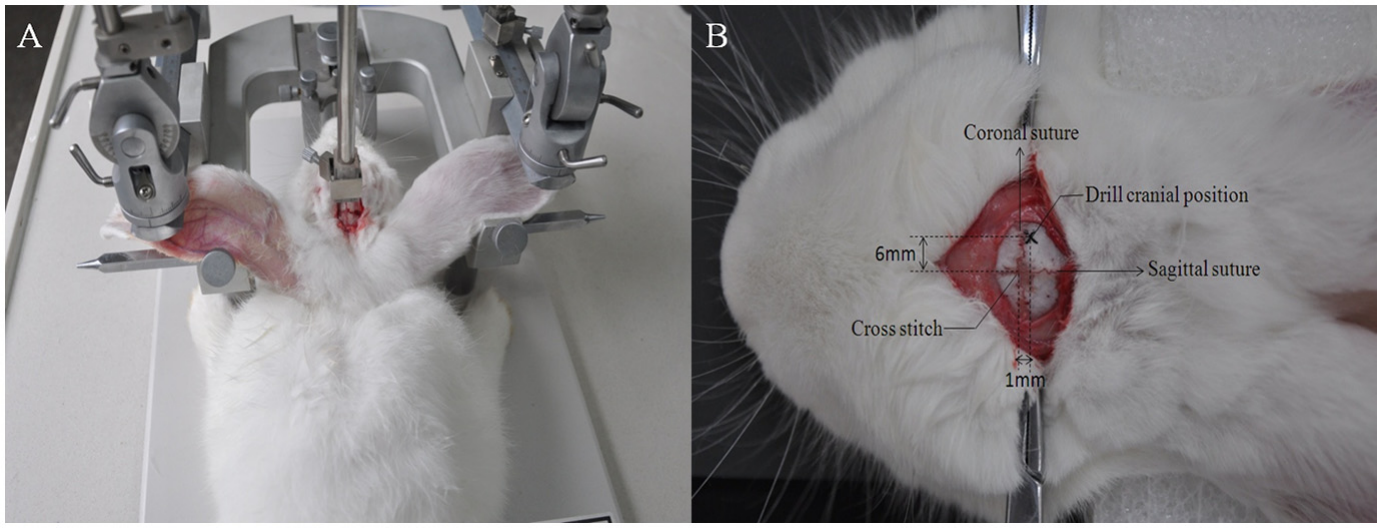
Determining position of drilling hole with stereotaxic instrument(A). Schematic of drill hole(B).

The rabbit cerebral hemorrhage model was established by the autologous blood injection method [26]. The model started with anesthetization via ear injection of urethane (25%, 5 ml/kg). A total of 4 ml blood was extracted from the femoral vein, after which a stereotaxic instrument (ZH-LanXingD, HuaibeiZhenghua Bio Equipment Co., Ltd., China) was used to perform cranial drilling. The brain “cross stitch” intersection was set as the starting point, and a puncture needle point was inserted 6 mm to the right of the coronal suture, 1mm posterior to the sagittal suture (Fig 2). A needle tube with a diameter of 0.7 mm was inserted into the brain to a depth of 13 mm. Dental cement was used to seal the crack between the needle tube and cranial hole to prevent the injected blood flowing along the needle.

Different states of brain hemorrhage were simulated by controlling the amount of blood (step-wise increments of 1ml blood were injected at the rate of 0.5 ml / min). The rabbit cerebral hemorrhage model simulated five states: pre-operative condition, 0 ml of cerebral hemorrhage, 1 ml of cerebral hemorrhage, 2 ml of cerebral hemorrhage and 3 ml of cerebral hemorrhage.

#### 3.2. MIPSS measurements

First, the MIPSS of the healthy rabbit under anesthesia was measured before the cranial drilling surgery. Next, the MIPSS of the four model states (including 0 ml, 1 ml, 2 ml, 3 ml) were measured after cranial drilling surgery. The state of 0 ml included two measurement sections (post-operative phase shift baseline section and 0 ml phase shift measurement section). The states of 1 ml, 2 ml, and 3 ml executed one measurement section, respectively. A section took 5 min 22 sto complete 20 measurements. The average value of the 20 measurements was taken as the phase shift.

During the measurement process, the rabbits’ heads and the locations of the two coils were stationary. The temperature was controlled to within 22°C–24°C, and the humidity around 50%. Since rabbits may exhibit other reactions following cranial drilling surgery, we performed the measurement as soon as possible after surgery. After completion of the MIPSS measurements, 1 ml of 1.5 mol/L KCL solution was injected via ear vein to euthanize the rabbits.

#### 3.3. MR images of cerebrospinal fluid

The maximum conductivity of the cerebrospinal fluid (CSF) was 1.7 times the conductivity of the blood, 4 times that of the gray matter, and 7 times that of the white matter at frequency of 65.5647 MHz (see Table 1). Hence we think that the CSF parameters which changed during cerebral hemorrhage had the greatest effect on the parameters. In order to better verify and explain the results, MR imaging of the rabbit head was performed, which showed the changes in the CSF of the five cerebral hemorrhage states. The steps of MRI measurements are same with the steps of MIPSS measurements, and using the same approach to euthanize rabbits after the experiment was completed.

**Table 1:** Electricalconductivity of brain tissues at frequency of 65.5647 MHz^a^.

Tissue name	Conductivity[S/m]
Cerebrospinal Fluid	2.0687
Blood	1.2083
Brain Grey Matter	0.5139
Brain White Matter	0.2936

^a^The data refer to Italian National Research Council Institute for Applied Physics, Calculation of the Dielectric Properties of Body Tissues in the frequency range 10 Hz-100GHz.

CSF MRI was performed using a 3.0T MRI scanner (Magnetom Spectra with A Tim + Dot System, Siemens, Germany) and an extremity 18 knee coil in Southwest Hospital, Chongqing, China. CSF MR images acquisition utilized SPACE (Sampling Perfection with Application optimized Contrast using different flip-angle Evolution) sequence. SPACE was T2-weighted turbo-spin-echo sequence, which can be clearly distinguished CSF from the surrounding tissue. MRI scanner parameters were set as follows: TR = 1300 ms, ETL = 49, TE = 44 ms, matrix = 320 × 275, FOV = 160 mm × 160 mm, number of slices = 192, slice thickness = 0.5 mm, slice spacing = 0 mm total scanning time = 5 min 22 s.

### 4. Experimental Data Analysis and Processing

To demonstrate the significant difference of MIPSS under the five cerebral hemorrhage states, a non-parametric statistical multiple comparison rank sum test (Friedman M Test) was utilized for the MIPSS data analysis. The statistical analysis utilized the program SPSS12.0 (SPSS Inc.) and the significance level criterion was p<0.05.

**Fig 3:**
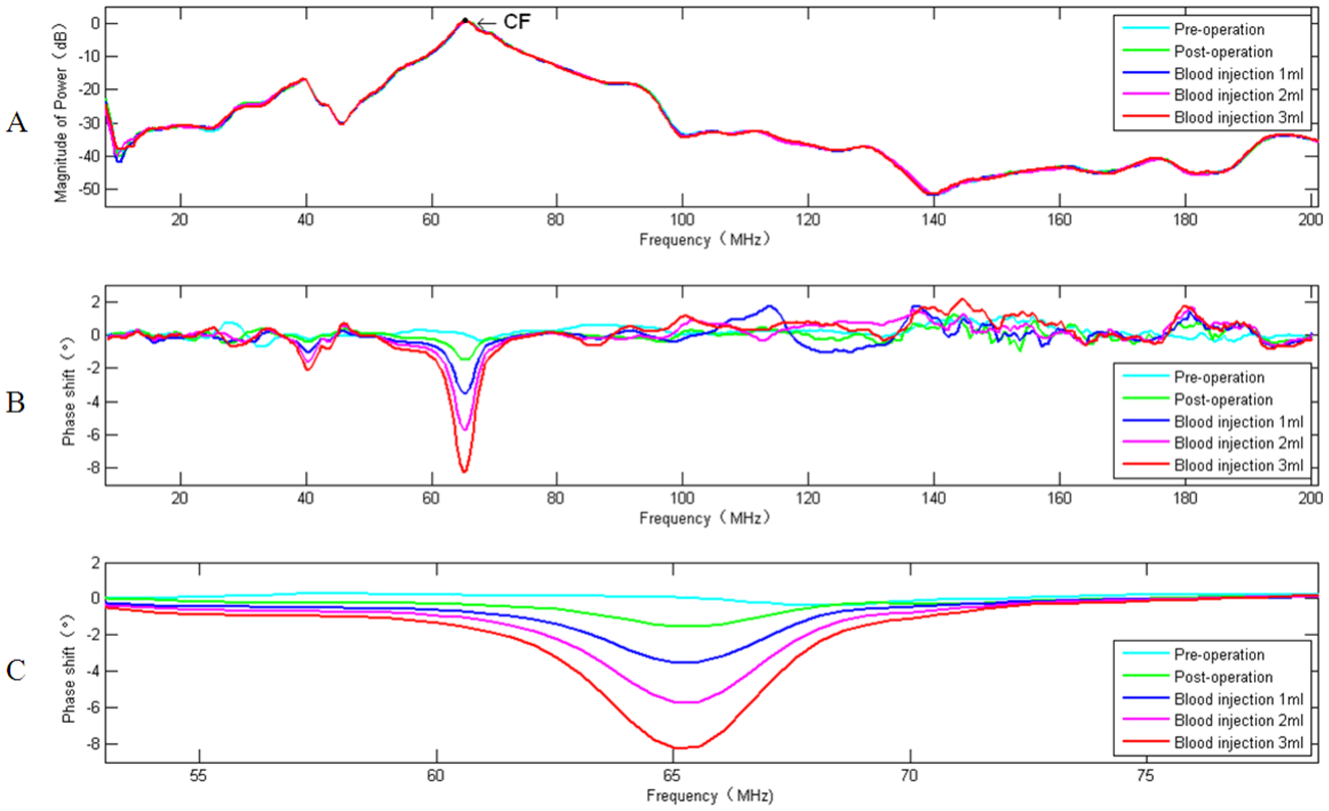
S_21_ power amplitude of experimental system from 1MHz to 200MHz (A). CF = 65.5647 MHz, its corresponding power values marked on the curve. Experimental measurements of phase shift as a function of frequency (1–200 MHz) for five volumes of autologous blood injected into brain of rabbit (B). The MIPSS of cerebral hemorrhage in rabbit under FB (53.0520 MHz–78.5776 MHz) (C). (A)-(C) frequency is on a linear scale. (B)-(C) data are shown as homogenized values of the 20 measurements with respect to baseline data.

According to the characteristics of the cerebral hemorrhage phase shift spectroscopy under FB, as shown in Fig 3C, an effective method for assessing cerebral hemorrhage severity reflects the severity of a single sample of cerebral hemorrhage and its overall trends. The phase shift spectroscopy under FB was divided into two sections: pre-characteristic frequency and post-characteristic frequency. Five phase shifts were taken at equal intervals from each section, the sum of five phase shift data of former section and latter section were defined as B_*ij*_ and F_*ij*_, respectively. *i* represented the *i*-th sample, *i*∈{1,2,3…13}; *j* represented the blood injection volume, *j*∈{+0,0,1,2,3}, and the "+" sign indicated status before cranial drilling surgery. The correction coefficient *K*_*ij*_ was defined as follows:

$$K_{ij}=V_{sj}/V_{ij}$$ (2)

*V*_*ij*_ was the phase shift value corresponding to *j* ml volume of blood injection of the *i*-th sample under characteristic frequency, and *V*_*sj*_was the average value of *V*_*ij*_ of all the samples.

$\bar{B_{ij}}$ value and $\bar{F_{ij}}$ value were obtained by multiplying the correction coefficient *K*_*ij*_ with *B*_*ij*_ and *F*_*ij*_.

$$\bar{B_{ij}}=K_{ij}*B_{ij}$$ (3)

$$\bar{F_{ij}}=K_{ij}*F_{ij}$$ (4)

The *B-F* distribution profile was drawn by taking $\bar{B_{ij}}$ value as the horizontal axis and $\bar{F_{ij}}$ value as the vertical axis in the rectangular coordinate. In the *B-F* profile, the locations around the coordinate indicated the pre-operation state, and the points at greater distance represented a more severe degree of cerebral hemorrhage. The formulas above showed that a larger sample was associated with a more accurate $\bar{B_{ij}}$ and $\bar{F_{ij}}$ correction value. Hence the *B-F* distribution profile was more meaningful.

## Results

In one of the animals, the ICP from 15mmHg rose to 52mmHg, and the HRV decreased markedly from 300 to 226 after the injection of 3-ml of blood. We selected this rabbit's results as an analysis example. Fig 3A showed the relationship between the power amplitude of cerebral bleeding volumes and frequency, with the five states of the power amplitude spectrum almost overlapping, thus indicating that the power amplitude was insensitive to the change in brain hemorrhage; Fig 3B showed the relationship between the phase shift of cerebral bleeding volumes and frequency, which was detected by the cerebral hemorrhage MIPSS method. The results of Fig 3A and 3B showed that the amplitude of the transmission parameter S21 was closely related to the detection sensitivity of the phase shift. A higher power of the frequency band corresponded to a greater phase shift. By using the power amplitude information to define the peak power point, its corresponding frequency was defined as the characteristic frequency (CF). The MIPSS under FB with the highest detection sensitivity and the greatest stability was selected (Fig 3C), which reflected the phase shift spectroscopy of each level of cerebral hemorrhage. Compared to the preoperative phase shift spectroscopy, after drawing blood and performing cranial drilling surgery, excursion occurred in the phase shift spectroscopy when blood injection was not applied. The phase shift increased with the successive increase of blood injection volume, indicating a positive relationship. This finding is similar to the results reported by Jin G, et al. [21–23] and Gonzalez et al. [27].

The non-parametric statistical Friedman M test was applied to the MIPSS data analysis. The highlight of the analysis is displayed in Table 2. The non-parametric statistical Friedman M test detected statistically significant differences among the five states of cerebral hemorrhage MIPSS measurements, with a significance level of *P*<0.05, and the frequency ranged from CF-5 MHz to CF+5 MHz. The test results showed statistically significant differences among the MIPSS data of the five states of cerebral hemorrhage under FB.

**Table 2:** Statistical analysis with a Friedman M test of the MIPSS data for the 5 states of cerebral hemorrhage under the FB in which a statistically significant difference of P<0.05 was found.

Freq. (MHz)	Ranks					p-level	Valid N
	Pre-operation	Post-operation	1 ml	2 ml	3 ml		
CF-5	4.23	4.54	3.23	1.92	1.08	2.81e-9	13
CF-4	4.23	4.54	3.23	2.00	1.00	1.97e-9	13
CF-3	4.23	4.54	3.23	2.00	1.00	1.97e-9	13
CF-2	4.46	4.54	3.00	2.00	1.00	4.78e-10	13
CF-1	4.54	4.46	3.00	2.00	1.00	4.78e-10	13
CF	4.54	4.46	3.00	2.00	1.00	4.78e-10	13
CF+1	4.54	4.46	3.00	2.00	1.00	4.78e-10	13
CF+2	4.15	4.47	3.08	2.00	1.00	5.23e-10	13
CF+3	4.00	4.77	3.23	2.00	1.00	1.06e-9	13
CF+4	3.62	4.77	3.23	2.15	1.23	8.74e-8	13
CF+5	3.54	4.31	3.08	2.15	1.92	0.000459	13

**Fig 4:**
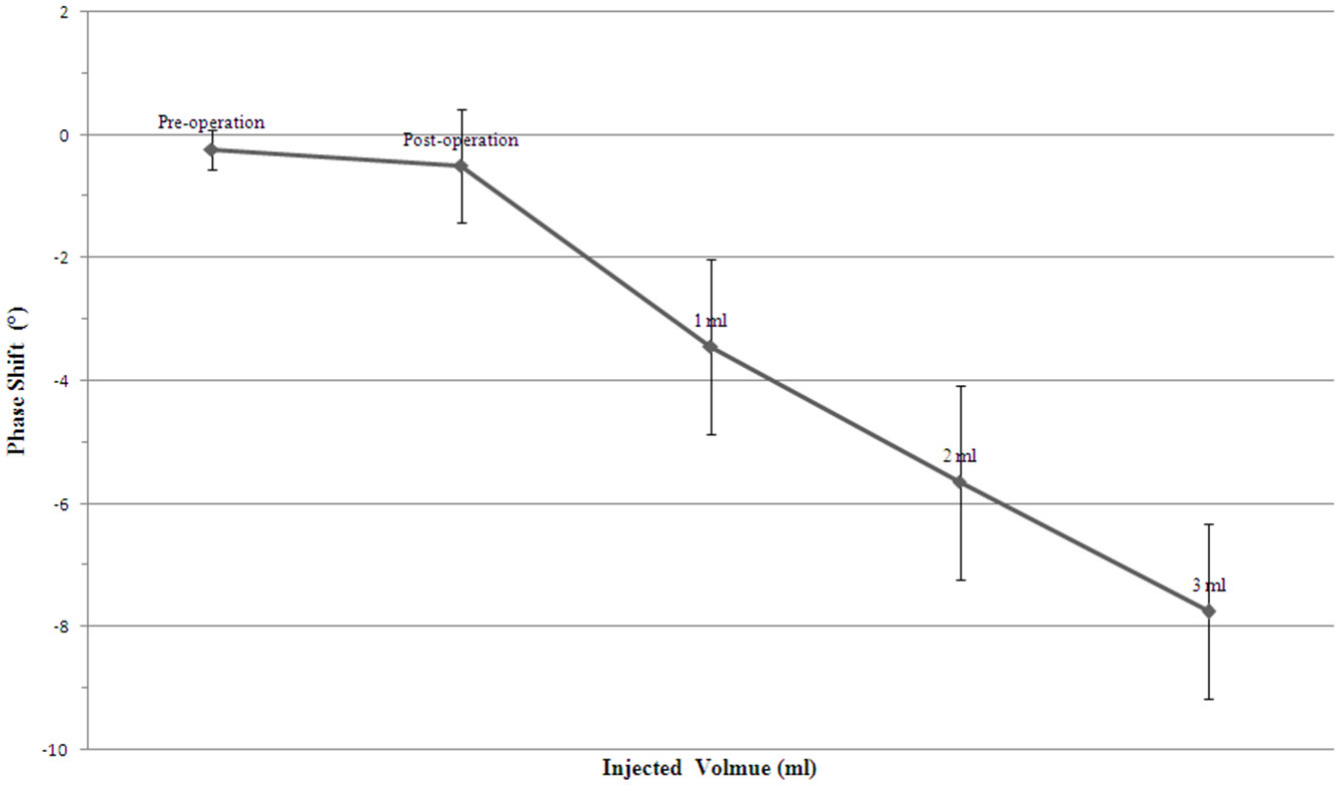
Average phase shift as a function of injected volumes at CF. Data are shown as homogenized values with respect to baseline data. Error bars indicate standard error from 13 animals.

Average phase shift data were taken from 13 rabbits with 5 states of cerebral hemorrhage at the CF (see Fig 4). The average phase shift of 3 ml of brain hemorrhage reached -7.7503°±1.4204°. Its sensitivity was increased by 12.5-fold when compared with the previous work with the single coil—coil method in which the frequency of the excitation signal was 10.7 MHz and the average phase shift of 3 ml of brain hemorrhage was 0.6173° ± 0.1976°[22]. Moreover, its sensitivity was increased by 4-fold when compared with symmetric cancellation-type sensor detection method based on the symmetry between the two brain hemispheres in which the excitation frequency was 7.5 MHz and the average phase shift of 3 ml of brain hemorrhage was 1.885° ± 0.242°[23]. The average phase shift caused by pre-operation, post-operation, 1ml injection and 2 ml injection were -0.2373°±0.3126°, -0.5031°±0.9257°, -3.4449°±1.4208° and -5.6422°±1.5761°, respectively. Details of 5 levels of the cerebral hemorrhages of 13 animals are shown in Table 3. It should be noted that, compared with the pre-operative phase shift, the post-operative phase shift difference produced a change of 0.2658°, due to the small amount of blood after cranial drilling surgery. It should also be noted that the first, second and third injections of 1 ml of blood produced phase shift difference changes of 2.9418°, 2.1973° and 2.1081°, respectively. This indicates that the relative phase shift between two consecutive injection states is decreased by a larger volume of blood present in the brain.

**Table 3:** Descriptive statistics (13 rabbits) of 5 states cerebral hemorrhage phase shift data at CF.

Cerebral hemorrhage State	N	Mean	Std. Deviation	Minimum	Maximum
Pre-operation	13	-.2373°	.3126	-.6553	.4648
Post-operation	13	-.5031°	.9257	-2.5652	.7887
Blood injection1 ml	13	-3.4449°	1.4208	-5.7004	-.9208
Blood injection 2 ml	13	-5.6422°	1.5761	-8.7272	-3.8029
Blood injection 3 ml	13	-7.7503°	1.4204	-10.1800	-6.1536

**Fig 5:**
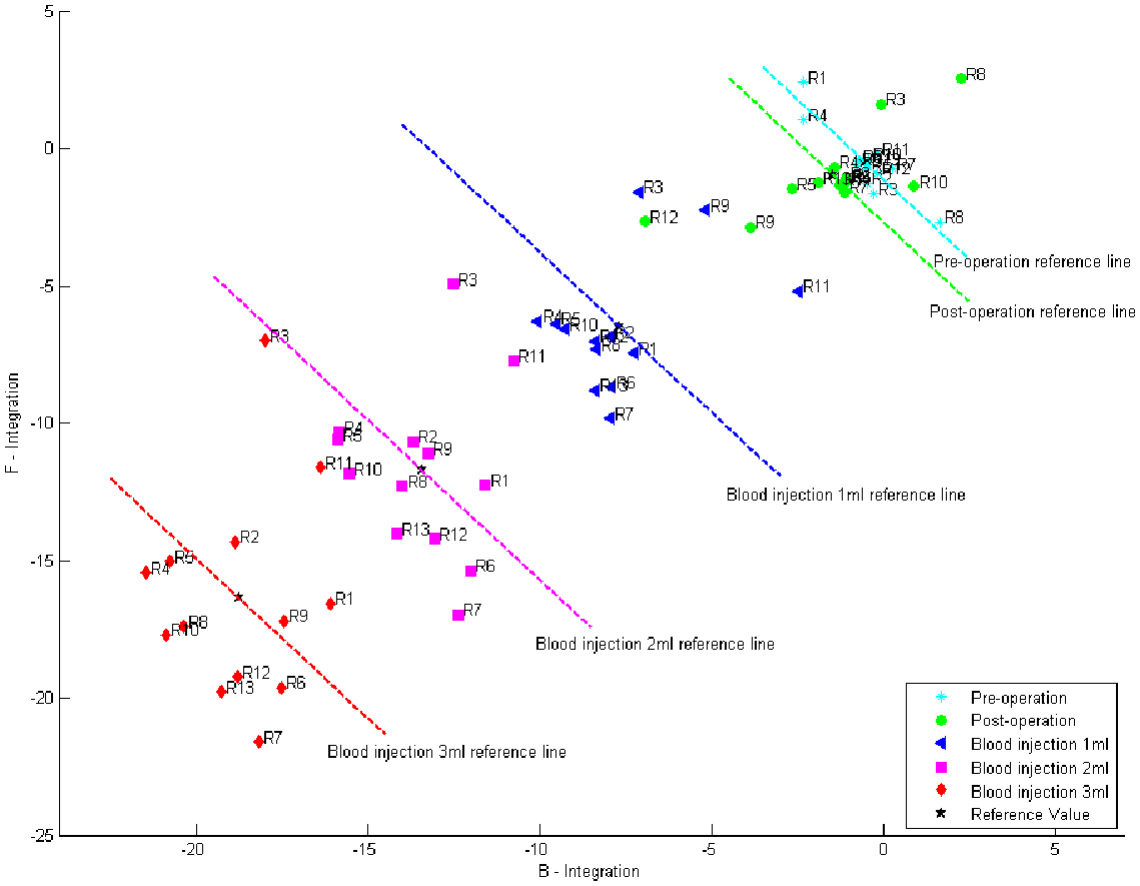
*B–F* distribution profile of 13 cerebral hemorrhage rabbits. Each rabbit had five cerebral hemorrhage levels, a data point corresponding to a cerebral hemorrhage level of one rabbit, for a total of 65 sets of B-F values. Reference value and reference line of each level were calculated from the average data of 13 test samples. Ri representative experiment serial number (i = 1,2,…13).

Based on the phase shift spectroscopy characteristics under the FB, we designed a *B-F* distribution profile to diagnose the severity of cerebral hemorrhage. Fig 5 is the *B-F* distribution profile of the cerebral hemorrhages of 13 rabbits with the FB-based MIPSS detection method. In the *B-F* distribution profiles, the pre-operative state distributes around the coordinate origin (0, 0), and with the increase in amount of cerebral hemorrhage, the *B-F* distribution moves farther away. In Fig 5, the distance of the reference line indirectly reflects the relationship between the injection volume of blood and rabbit head characteristic parameters (mainly conductivity). The distance between the reference line decreases with the increase of the injection volume of blood, which indicates that the increase in the injection volume of blood caused a smaller variation of the rabbit head characteristic parameter. This finding is consistent with the phase shift trend in Fig 4.

**Fig 6:**
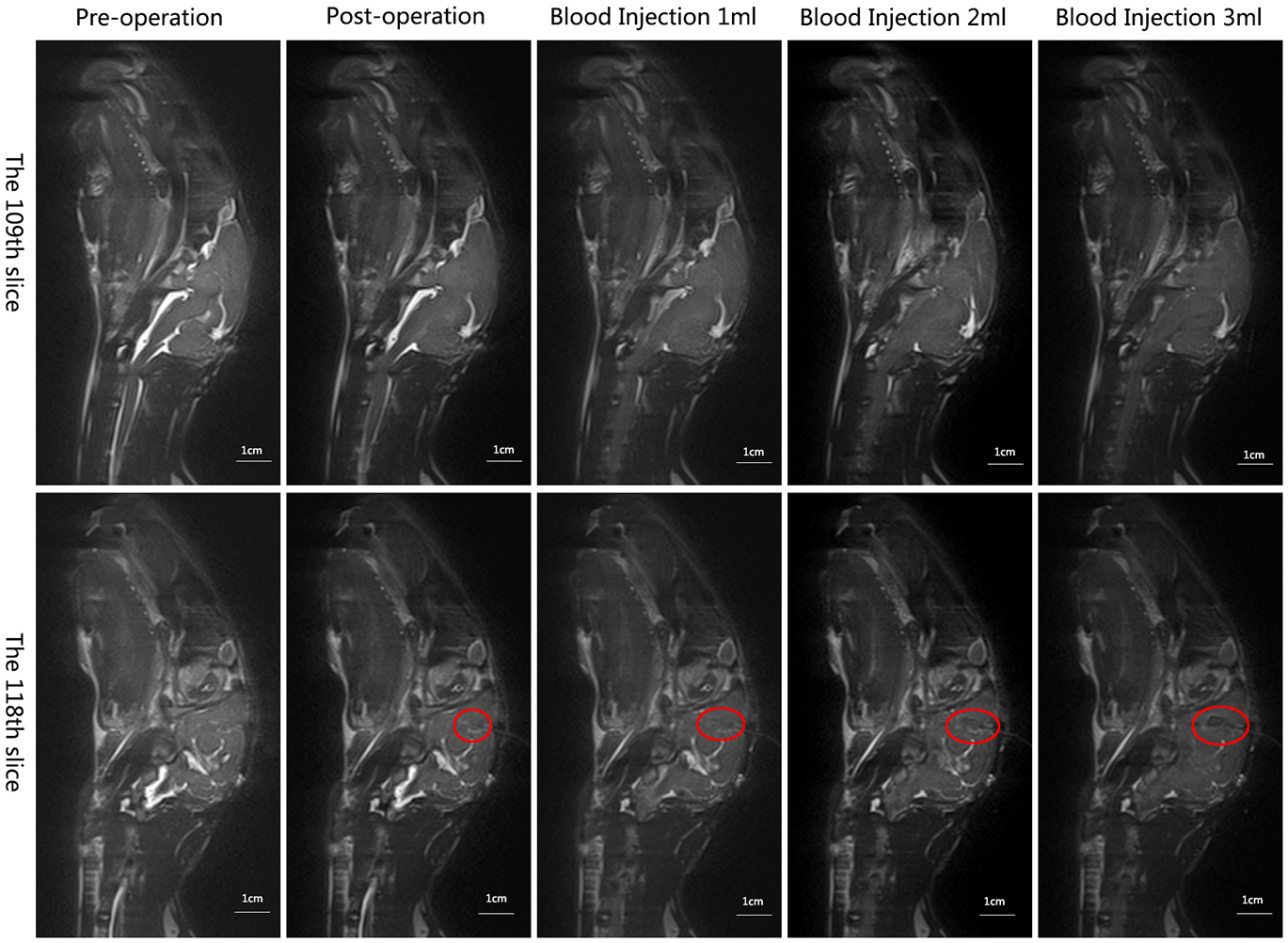
MRI of CSF changed by injecting blood at sagittal plane in a rabbit head. Cerebrospinal fluid is displayed as highlighted signal. The injection position is circled in red. The upper row is the 109th slice, and the bottom row is the 118th slice. Left to right: pre-operative, post-operative (0 ml), blood injection 1 ml, blood injection 2 ml, and blood injection 3 ml.

MRI of the rabbit head was performed to show the changes in the CSF of the five cerebral hemorrhage states. Fig 6 shows the CSF changes resulting from injecting blood into the rabbit’s head. With the blood injection volume increased, there was a more obvious decrease in CSF. CSF without injected blood underwent minor changes post-operatively compared with its pre-operative condition. The most obvious change in CSF occurred between 0 ml (without injected blood) and 1 ml, and there are slight differences in CSF between 2 ml and 3 ml. These images show that there is a continuous discharge of CSF from 0 to 2 ml, approaching the maximum volume after 2–3 ml, indicating that the changes of the conductivity became smaller with the increase of blood injection volume, which is consistent with the phase shift trend in Fig 4 and the overall trend of *B-F* distribution in the rabbit cerebral hemorrhage in Fig 5.

## Discussion

The coil structure (single coil-coil) used in this study determined that the measured phase shift reflected the changes of the whole rabbit brain conductivity. The change of conductivity of the whole brain was related to physical adjustment and physiological regulation when a cerebral hemorrhage occured, which was caused by a combination of factors. The blood was injected into rabbits’ heads to cause changes in brain tissue parameters, which were associated with the injection volume and the dynamic changes of other brain tissue fluids. Since the cranial cavity can be considered to be a rigid container with its volume unchanged, the injected blood would be expected to squeeze out other brain tissue fluid [28], especially the CSF (the maximum conductivity part in the brain tissue). From the perspective of physiological regulation, the reduction in CSF was related to its secretion inhibition and its accelerated metabolism in the process of cerebral hemorrhage [29]. In this study, MR imaging of the rabbit head showing the changes in CSF of the five cerebral hemorrhage states was performed, in order to verify and interpret the MIPSS results. The changes of CSF and the results of MIPSS showed a certain degree of correlation that indicated the validity of our experimental results. However, this research only preliminarily studied the change in the CSF caused by cerebral hemorrhage, without studying the changes of other tissues, and more comprehensive and in-depth research needs to be conducted.

As discussed above, the purpose of this study was to measure the change in the conductivity of a rabbit brain. The greater the conductivity changes, the greater the phase shift generated. The complex impedance of biological tissue, in the frequency range from DC through MHz to GHz, can be divided into three main dielectric dispersions [30]. The electrical permittivity and conductivity of the three main dielectric dispersions have been labeled α, β and γ, which represents the frequency range of 1 Hz- kHz, 0.1–100 MHz and 0.1–100 GHz, respectively. The α-dispersion is caused by the relaxation in the counter-ion atmosphere surrounding the charged cell membrane surface; the β-dispersion is produced by Maxwell—Wagner effects, an interfacial relaxation process occurring in materials containing boundaries between two different dielectrics; and the γ-dispersion is caused by the relaxation of free water within tissues [31]. Since the rabbit brain is a complex electrical conductor constructed by a number of tissue materials with different dielectric properties, the conductivity changes caused by the brain tissue interfacial relaxation should be maximum under FB. During the injection of blood into the rabbits’ heads, different levels of brain tissues deform under different pressures because of the uneven distribution of stress. This changes the dielectric properties of different interface tissues, increasing the tissue interface, the interfacial relaxation effects, and the conductivity variation, thus resulting in a greater phase shift.

As can be seen in Fig 3A, five cerebral hemorrhage states of power amplitude spectrum almost overlap. This indicates that while the power amplitude is insensitive to the change in brain hemorrhage, the phase shift shows more sensitivity. Furthermore, Fig 3A and 3B shows that at the CF, the response amplitude of the power transmission parameter S_21_ is maximum, corresponding to the biggest phase shift of the cerebral hemorrhage. This indicates that the magnetic energy coupling within the two coils, with frequency-dependent properties, has a greater impact on the sensitivity of the detection system. An equivalent circuit diagram of the magnetic induction detection for cerebral hemorrhage is shown in Fig 1B. It can be equivalent to two RL series circuits [32],which contain the source impedance (Z_g_), transmission line impedance (Z_L1_ and Z_L2_) and load impedance (Z_exc_ and Z_ind_). The impedance matching between circuit elements is particularly important at higher frequencies, which determines the sensitivity of the detection system and the accuracy of the experimental results. Coil impedance varies with frequency, which approximatively matches the characteristic impedance of the coaxial line at the CF. With the impedance matching between circuit elements, the excitation signal is in the state of a traveling wave, allowing the most accurate measurement results. At the same time, the power transfer efficiency of the detection system is maximum, and the stronger main magnetic field stimulates the stronger disturbance magnetic field of the brain lesions, resulting in a greater measured phase shift. In addition, the stronger the magnetic field energy, the better the system stability and anti-jamming capability.

The results of MIPSS data analysis and processing under the FB, shown in Table 2, Table 3, Fig 4 and Fig 5, fully demonstrate the feasibility of using the MIPSS method to measure cerebral hemorrhage in rabbits. The MIPSS data of whole band shows that MIPS detection sensitivity has frequency-dependent properties, which indicates that the operating frequency of the excitation signal is particularly important while using the MIPS detection method to measure cerebral hemorrhage, and that the CF with the highest detection sensitivity and the greatest stability should be selected so as to ensure good results detected. It should be noted that the use of different coils obtained different MIPSS results. This is because the circuit parameters were not the same, and show different FB. It is certain that, no matter which coils are used, the sensitivity of cerebral hemorrhage detection system is highest at CF, and the most accurate results are measured under FB.

The cerebral hemorrhage B-F distribution is a typical two-parameter scalar collator, which can provide instantaneous diagnostic information about the severity of the cerebral hemorrhage from a single set of measurements. A similar two-parameter scalar classifier appears in Gonzalez et al. [10]. They diagnosed the condition of the brain, including healthy volunteers and patients with hemorrhage and edema, as a function of two volumetric electromagnetic phase-shift spectroscopy (VEPS) parameters in the β and γ ranges of frequency. Apparently, the *B-F* distribution profile is able not only to clearly distinguish the pre-operative state and various levels of cerebral hemorrhage but also to reflect the overall trend of the cerebral hemorrhage. We can determine the severity of cerebral hemorrhage and its progression according to the two-dimensional coordinate information. In Fig 5, the valid point represents the information deviation, which is due to the individual differences of each rabbit and the surgical procedure error.

This study used a single coil-coil to detect the time-difference MIPSS between before- and after- haemorrhage states and between two haemorrhagic states with different volumes of blood. Although the time-difference phase shift may allow the possibility of monitoring the progression of a hemorrhagic stroke, it is unlikely to be of use in making an initial diagnosis since a before-stroke data will not be available in clinic [11]. The frequency-difference method, which depends on the ability to distinguish the ‘frequency signature’ of a lesion from that of the surrounding tissues, is being explored to overcome this limitation. However, the phase shift of the hemorrhage by this method is significantly more difficult given that the conductivity of all of the tissues of the head changes with the frequency [13]. The results of this study show that the MIPSS detection method is able to provide a new possibility for real-time monitoring and diagnosis of the severity of cerebral hemorrhage. It is known from clinical studies that the changes in the cerebral hemorrhage are complex. Therefore more comprehensive and in-depth research needs to be conducted in our future work.

## Conclusion

This study employed the MIPSS method for detecting cerebral hemorrhage in rabbits. A rabbit model of cerebral hemorrhage was studied within 1MHz- 200MHz, and the optimal feature band was selected for analysis. When compared with a conventional single coil-coil detection method [22], and the method of a symmetric cancellation-type sensor detection system [23], the sensitivity of the FB-based MIPSS increases by 12.5 times and 4 times. Furthermore, the *B-F* profile design based on the MIPSS method can effectively distinguish the degree of severity of cerebral hemorrhage, showing the potential application value of the MIPSS detection technology. Since this study is mainly based on animal experiments, its clinical application requires more in-depth study based on animal experiments and clinical research, so as to improve the validity and reliability of the MIPSS method for detecting cerebral hemorrhage.
